# Risk factors for fecal carriage of carbapenemase producing Enterobacteriaceae among intensive care unit patients from a tertiary care center in India

**DOI:** 10.1186/s12866-016-0763-y

**Published:** 2016-07-08

**Authors:** Gajanand Mittal, Rajni Gaind, Deepak Kumar, Gaurav Kaushik, Kunj Bihari Gupta, P. K. Verma, Monorama Deb

**Affiliations:** Department of Microbiology, VMMC and Safdarjung Hospital, New Delhi, 110029 India; Department of Anaesthesia, VMMC and Safdarjung Hospital, New Delhi, 110029 India

**Keywords:** Risk factor, Carbapenem resistant Enterobacteriaceae, Carbapenemase producing Enterobacteriaceae, Gut colonization, ESBL, AmpC, Intensive care unit

## Abstract

**Background:**

Resistance amongst the commensal flora is a serious threat because a very highly populated ecosystem like the gut, may at a later stage, be a source of extra intestinal infections, resistant strains may spread to other host or transfer genetic resistance element to other members of micro-biota including pathogens. This study was carried out to assess fecal colonization by carbapenemase producing Enterobacteriaceae (CPE) and associated risk factors among 100 patients admitted to intensive care unit (ICU). The phenotypic and molecular characterizations of CPE were also included.

**Results:**

Colonization with CPE was observed in 6.6 % (8/122) controls. Among ICU patients, fecal carriage of CPE was significantly higher on day 4 (D4) (22 %) as compared to day 1 (D1) (11 %) (*p* value 0.002). The carbapenemase genes detected included OXA- 48, 181, KPC and NDM-1 with NDM-1 being the predominant carbapenemase in both ICU D1 and D4. Among the 50 CPE isolates, 8 (16 %) were susceptible to meropenem and imipenem (Minimum inhibitory concentration; MIC ≤ 1 mg/L) and all were susceptible to colistin (MIC range 0.125 - 1 mg/L) and tigecycline (MIC range 0.06- 1.5 mg/L).

The risk factors associated with CPE carriage were duration of ICU stay, use of ventilator and aminoglycosides.

**Conclusions:**

Prior colonization with CPE could result in their influx and spread in ICU, challenging infection control measures. Exposure to ICU further increases risk of colonization with diverse carbapenemase-producing Enterobacteriaceae. Gut colonization with these strains may be a source of endogenous infection and horizontal transfer of these genes in future.

**Electronic supplementary material:**

The online version of this article (doi:10.1186/s12866-016-0763-y) contains supplementary material, which is available to authorized users.

## Background

Antibiotic resistance is observed in both pathogenic bacteria and normal commensal flora [1]. Current strategies to monitor antibiotic resistance mainly examine pathogenic organisms and only periodic cross-sectional evaluations of resistance are undertaken for commensal flora [2]. Members of family Enterobacteriaceae heavily colonize human gut and are the most frequent cause of bacterial infections in patients of all ages [3]. Their ubiquity and frequent acquisition of mobile genetic elements means that their human hosts are regularly exposed to new strains with novel genetic repertoires including antibiotic resistance through food, water, and from other animate and inanimate sources in the community [3]. Resistance amongst the commensal flora is a serious threat because a very highly populated ecosystem like the gut, may at a later stage, be a source of extra intestinal infections, resistant strains may spread to other host or transfer genetic resistance element to other members of micro-biota including pathogens [4].

Extended spectrum β-lactamase (ESBL) producing Enterobacteriaceae are widely disseminated in India and carbapenems are drug of choice for infections due to ESBL producers [5, 6].

As gastrointestinal tract may serve as a reservoir for carbapenem resistant Enterobacteriaceae (CRE), resulting in cross transmission in health care settings, active surveillance among high risk patients is necessary for prevention and control of their dissemination in acute care facilities [7]. In most surveillance studies perianal and rectal cultures were generally found to be most reliable for screening colonization with resistant Enterobacteriaceae [8]. Several studies have been conducted to assess the risk factors associated with colonization and infection caused by ESBL producing Enterobacteriaceae [9, 10]. There is paucity of data regarding fecal carriage of CRE.

The predominant mechanism of carbapenem resistance among Enterobacteriaceae is through acquisition of diverse carbapenemases belonging to class A, B and D. Recent reports suggest that NDM-1 (Class B, metallo-β-lactamase) is widely distributed among Enterobacteriaceae in the environmental samples and key enteric pathogens in India [6].

This study was undertaken to assess fecal colonization with carbapenemase producing Enterobacteriaceae (CPE) and associated risk factors among patients admitted to intensive care unit (ICU). Antibiotic susceptibility profile and molecular characteristics of these isolates were also studied.

## Methods

### Specimen collection

The study was conducted in a 10 bedded ICU from September 2011 and September 2012. Patients requiring intensive care or ventilator support were admitted directly to ICU from the emergency department or operation theatre and included patients of acute poisoning, neurological or cardiovascular emergency, acute respiratory failure and patients undergoing emergency and elective surgery. Patients transferred to the ICU from other wards and/or hospitals were excluded. Two rectal swabs were collected from all enrolled patients (*n* = 100), one each on day 1 (D1) and day 4 (D4) of ICU admission and transported in nylon flocked system in Amies liquid transport medium. Patients, in whom D4 sample could not be collected, were excluded from the study. To study risk factors for colonization; demographic data, clinical history, current antibiotic therapy, including carbapenems, invasive procedures and co-morbid conditions were recorded through chart review in a predesigned proforma (Additional file 1). Controls included, stool samples from 122 non-hospitalized patients attending the outpatient department for routine health checkup. This study was approved by the Institute Ethics Committee, V.M.M.C & Safdarjung Hospital (S.NO. VMMC/SJH/Ethics/SEP-11/29).

### Isolation and identification

Approximately 0.5gm of stool or rectal swab was emulsified in 0.5 ml of sterile 0.85 % saline. Screening of CRE was done on in-house prepared media by four different methods and included: (i) overnight selective enrichment in 5 ml tryptic soy broth with a 10 μg ertapenem disk followed by plating onto MacConkey agar (CDC protocol) [11], (ii) direct plating onto MacConkey agar supplemented with imipenem at 1 mg/L (MacI), (iii) direct plating onto MacConkey agar supplemented with cefotaxime at 1 mg/L (MacC ESBL), (iv) MacConkey agar with standard imipenem, meropenem and ertapenem Disk (10 μg Disk), applied at the 4-, 8-, and 12-o’clock positions (MacD). Evaluation of bacterial growth was made after 18 to 24 h of incubation at 37 °C in ambient air. All morphologically distinct colonies were sub cultured on standard MacConkey agar plate and isolates were identified by conventional biochemical tests [12]. The media used in the study had been previously validated for isolation of CRE harbouring carbapenemase genes (KPC, IMP, VIM, NDM-1, OXA-48 and OXA-181).

### Susceptibility testing and phenotypic detection of β-lactamases

Antibiotic susceptibility was performed by disk diffusion as per clinical and laboratory standards institute (CLSI) 2012 guidelines [13] for ampicillin (10 μg), trimethoprim-sulfamethoxazole (1.25/23.75 μg), piperacillin (100 μg), ceftazidime (30 μg), cefotaxime (30 μg) cefoxitin (30 μg), piperacillin-tazobactam (100/10 μg), cefoperazone-sulbactam (75/30 μg), netilmicin (30 μg), amikacin (30 μg), gentamycin (10 μg), nalidixic acid (30 μg), ciprofloxacin (5 μg), tetracycline (30 μg). All Enterobacteriaceae were screened for carbapenem resistance using ertapenem (10 μg), meropenem (10 μg), imipenem (10 μg) disk and results were interpreted as per CLSI 2012 guidelines. All isolates with ertapenem zone diameter <22 mm were subjected to minimum inhibitory concentration (MIC) for ertapenem, meropenem, imipenem, colistin and tigecycline using E test (bioMérieux, France). Isolates with ertapenem MIC > 0.5 mg/L were defined as CRE. Resistant and susceptible to tigecycline were defined as MIC˃2 mg/L and ≤1 mg/L respectably (European committee on antimicrobial susceptibility testing; EUCAST 2011).

All CRE were screened for β-lactamases by phenotypic test and PCR. For phenotypic test commercially available KPC, MBL and ESBL/AmpC Screen kit (Rosco Diagnostica, Denmark) containing meropenem and cefotaxime alone and with various β-lactamase inhibitors (boronic acid, dipicolinic acid, clavulanic acid, cloxacillin and clavulanic acid + cloxacillin) were used. Zone diameters were recorded. Tests were interpreted as per manufacture instruction.

### PCR for β-lactamases among CRE

DNA was isolated by phenol chloroform method as per Sambrook et al. [14]. PCR was performed for ESBL (SHV, TEM and CTX-M), AmpC (MOX, FOX, DHA, CIT, ACC, EBC) and Class A (KPC1 and 2), Class B (NDM-1, IMP, VIM) and class D (OXA- 48 and OXA-181) carbapenemases. PCR product were electrophoresed on 1.5 % agarose gel along with a 100 bp DNA ladder (as a molecular wt marker) and visualized using a UV transilluminator. For TEM [5], SHV [5], CTX-M [15], AmpC [16], NDM-1 [5], IMP [5], VIM [5], KPC [5] and OXA-181 [17] previously designed primers were used. For OXA–48, primer (self-deigned) used was FP (5′-GCGTGTATTAGCCTTATC-3′) and RP (5′-CGCGGTTCGGTAGTGTGTTT-3′), which amplifies a 760 bp product. Cycling conditions were: 95 °C for 5 min; 30 cycles of 95 °C for 30s, 60 °C for 30s and 72 °C for 60s; and 72 °C for 5 min. Selected amplicons were sequenced. All primers were procured from Integrated DNA Technologies (IDT).

CRE growing on selective media and positive for any carbapenemase gene were defined as carbapenemase producing Enterobacteriaceae (CPE). Non-CPE were defined as all CRE isolates growing on selective media and negative for any carbapenemase gene.

Controls used for antibiotic susceptibility, phenotypic test and PCR included ESBL-positive *Klebsiella pneumoniae* ATCC 700603, ESBL-negative *Escherichia coli* ATCC 25922, KPC-positive *Klebsiella pneumoniae* ATCC BAA-1705, KPC-negative *Klebsiella pneumoniae* ATCC BAA-1706. For IMP, VIM, NDM-1, OXA-48 and OXA-181 in house strains confirmed by sequencing were used as controls.

**Fig. 1:**
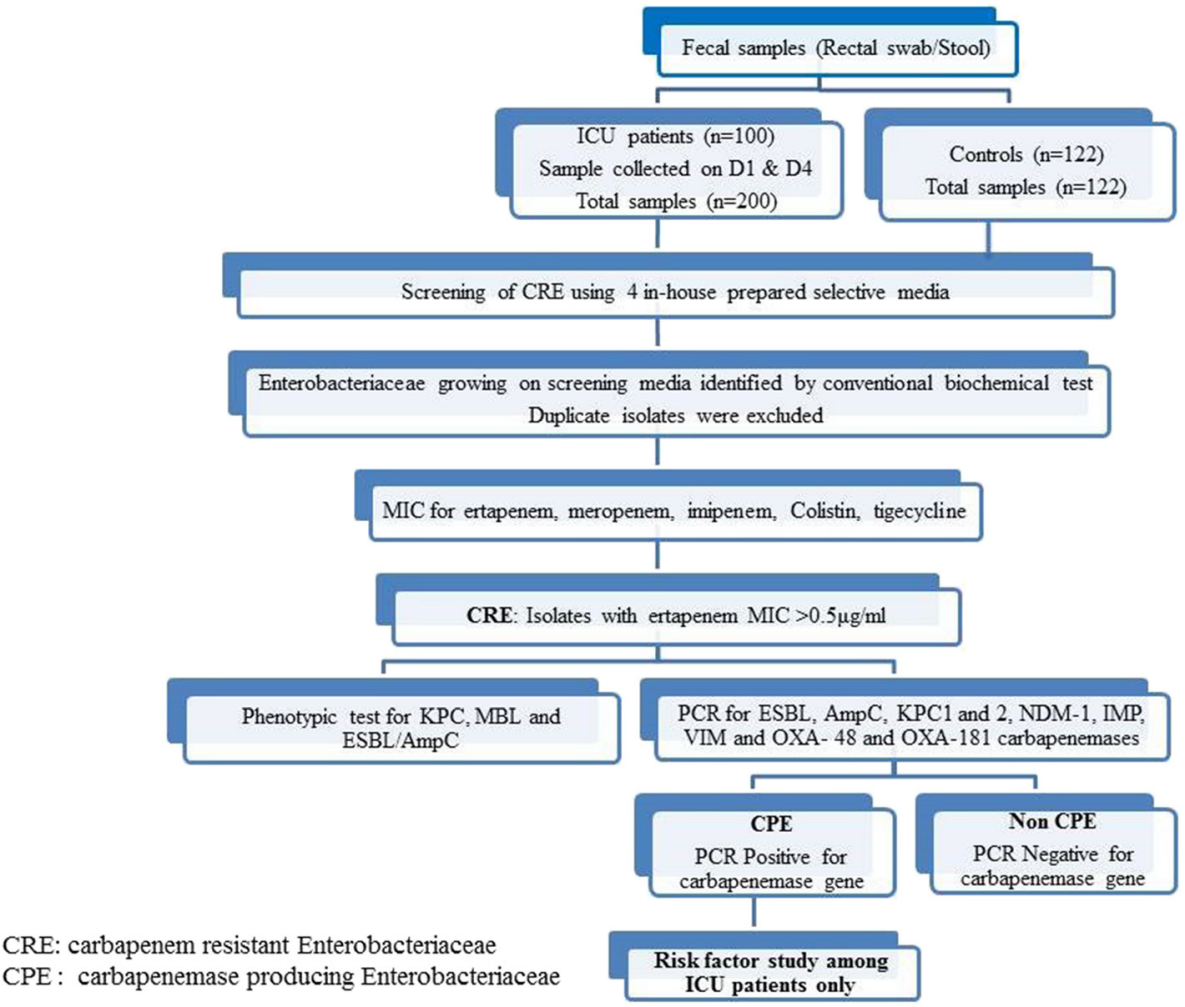
The flowchart of steps to study colonization with Carbapenemase producing Enterobacteriaceae (CPE)

Figure 1 shows the flowchart of steps to study colonization with CPE.

### Statistical analysis

Antibiotic resistance data was analyzed using WHONET 5.6. *χ*^2^ test was used for comparison of prevalence fecal carriage of CPE in ICU patients D1 vs D4 and ICU vs controls. Statistical significance was taken as *p* value <0.05.

Risk factors for colonization with CPE among ICU patients were compared using *χ*2 or Fisher exact test, as appropriate. All *p* values were two tailed. *p* value <0.05 was considered statistically significant. Adjusted odds ratios and 95 % confidence intervals (CIs) were computed for the significant factors. Variables that were present in more than 10 % of ICU patients with CPE colonization with *p* value <0.05 were entered into backward stepwise logistic regression models in multivariate analysis using SPSS version 20.0 (SPSS, Chicago, IL)

## Results

Isolation and characteristics of CRE and CPE from fecal samples are summarized in Tables 1 & 2. A total of 322 fecal samples were screened which included a single sample from 122 controls and 200 samples from 100 ICU patients, collected on D1 and D4. A total of 444 morphologically distinct Enterobacteriaceae were isolated from 89 (72.9 %) controls, 63 (63 %) and 58 (58 %) ICU patients on D1 and D4 respectively. Although 4 media were used for screening of CRE & CPE, duplicate isolates were excluded. Ertapenem MIC > 0.5 mg/L was observed among 91/444 (20.5 %) Enterobacteriaceae and were defined as CRE. Isolation of CRE was significantly higher among ICU patients on D4 (35/100, 35 %) compared to controls (14/122, 11.5 %) (*p* value 0.0001) and ICU-D1 (20/100, 20 %) (*p* value 0.02) (Table 1).

**Table 1:** Colonization pattern of CRE and CPE among controls and ICU patients

	Controls (%)	ICU	
		D1 (%)	D4 (%)
Total no. of patients screened	122	100	100
Total no. of patients with growth of Enterobacteriaceae	89 (72.9)	63 (63)	58 (58)
No of patients colonized with CRE	14 (11.5)	20 (20)	35 (35)**
No of patients colonized with CPE	8 (6.6)	11 (11)	22 (22)**

*CRE* carbapenem resistant Enterobacteriaceae, *CPE* carbapenemase producing Enterobacteriaceae, *Non CPE* non carbapenemase producing Enterobacteriaceae, *D1* Day 1, *D4* Day 4. ^**^ *p* value significant compared to ICU D1 (<0.05) and controls (<0.05)

**Table 2:** Phenotypic and molecular characteristics of CRE and CPE isolates from fecal samples

	Controls	ICU	
		D1	D4
Total Enteterobacteriaceae isolated (*n* = 444)	181	145	118
Characteristics of CRE (*n* = 91)	20	31	40
1. Species distribution pattern			
*Escherichia coli*	16	28	30
*Klebsiella sp.*	2	3	9
*Proteus sp.*	2	0	0
*Enterobacter sp.*	0	0	1
2. MIC range (mg/L)			
Ertapenem	0.75-64	0.75-64	0.75-64
Imipenem	0.25-64	0.25-64	0.25-64
Meropenem	0.064-64	0.047-64	0.064-64
3. Phenotypic characterization			
MBL	10	11	15
KPC	0	0	2
Non MBL/KPC Carbapenemase	0	3	9
Only ESBL and/or AmpC	10	15	11
No β lactamases class	0	2	3
Characteristics of CPE isolates (*n* = 50)	10	14	26
1. Species distribution pattern			
*Escherichia coli*	6*	14^¥^	19^$^
*Klebsiella sp.*	2*	0	6 ^ҍ^
*Proteus sp.*	2*	0	0
*Enterobacter sp.*	0	0	1^#^
2. MIC range (mg/L)			
Ertapenem	64-64	3-64	2-64
Imipenem	64-64	0.38-64	0.38-64
Meropenem	64-64	0.5-64	0.38-64
3. Distribution of β- lactamases			
TEM	4	10	14
SHV	1		4
CTXM	2	9	22
CIT	1		1
DHA	2	3	
MOX			2
NDM-1	10	12	17
KPC			2
OXA-48		1	7
OXA-181		2	8
Characteristics of NON-CPE isolates (*n* = 41)	10	17	14
1. Species distribution pattern			
*Escherichia coli*	10	14	11
*Klebsiella sp.*		3	3
2. MIC range (mg/L)			
Ertapenem	0.75-8	0.75-64	0.75-64
Imipenem	0.25-2	0.25-64	0.25-64
Meropenem	0.064-0.75	0.047-64	0.064-64
3. Distribution of β- lactamases			
TEM	5	9	9
CTXM	6	8	7
DHA			1
EBC		1	
CIT	5		
No enzyme detected	2	3	2

*CRE* carbapenem resistant Enterobacteriaceae, *CPE* carbapenemase producing Enterobacteriaceae, *Non CPE* non carbapenemase producing Enterobacteriaceae, *D1* Day 1, *D4* Day 4. *All NDM -1 (*n* = 10); ^¥^NDM-1 (*n* = 11), OXA-48(*n* = 1), OXA-48 + NDM-1(*n* = 1), OXA-181(*n* = 1); ^$^NDM-1(*n* = 12), OXA-48 + 181(*n* = 4), OXA-48(*n* = 1), OXA-48 + NDM-1(*n* = 1), OXA-48 + 181 + NDM-1(*n* = 1); ^ҍ^ NDM-1(*n* = 3), KPC (*n* = 1), OXA-48,181(*n* = 1), KPC + OXA-181(*n* = 1); ^#^OXA-181(*n* = 1)

Carbapenemase genes were detected among 50/91 CRE isolates and were defined as CPE. Colonization with CPE was observed among 6.6 % (8/122) controls as compared to 11 % (11/100) ICU patients on D1 (*p* value 0.3). Colonization increased to 22 % (22/100) on D4 of ICU stay (*p* value 0.001). Among 7 ICU patients CPE was present on both ICU-D1 and ICU-D4 (Table 1).

Carbapenemases were detected in diverse genera, *Escherichia coli* (39/50) was the predominant species in both ICU and control patients followed by *Proteus sp., Klebsiella sp.* and *Enterobacter sp*.

Among 91 CRE isolates synergy between meropenem with dipicolinic acid was observed among 36 CRE isolates and was predictive of presence of metallo-β-lactamases. Disks containing this combination generated zone diameters of at least 5 mm larger (mean 12 mm larger, range 5–19 mm) than those containing meropenem alone (10 μg); However the zones with meropenem plus dipicolinic acid were ≥27 mm in all but 3 isolates indicating coexistence of other non-metallo-β lactamases in the latter strains. Synergy with boronic acid, predictive of KPC or class A enzymes, was observed in 2 isolates. Synergy with clavulanic acid and or cloxacillin was observed in 36 CRE isolates and predicted presence of ESBL, AmpC or co-producers. 17 CRE isolates were negative for all phenotypic tests suggesting a presence of a class D carbapenemases or ESBL and/or AmpC with altered permeability due to efflux or changes in outer membrane porins. There was a good correlation between phenotypic test and PCR for carbapenemase genes. Isolates with OXA-48 and OXA-181 genotype showed no synergy with dipicolinic acid and boronic acid.

Amongst the controls, NDM-1 was the only carbapenemase gene detected. Amongst ICU patients carbapenemase genes were diverse (OXA-48, OXA-181 and KPC) with predominance of NDM-1. Diversity and number of coexisting carbapenemase genes increased with ICU stay (Table 2). NDM-1 co-existed with OXA-48, OXA-181 and KPC genotype co-existed with OXA-181. Among 50 CPE, 45 (90 %) were positive for ESBL genes, commonest being CTX-M 1. The predominant AmpC genes among CPE isolates were DHA, MOX and CIT. Details of these genes are shown in Table 2.

**Fig. 2:**
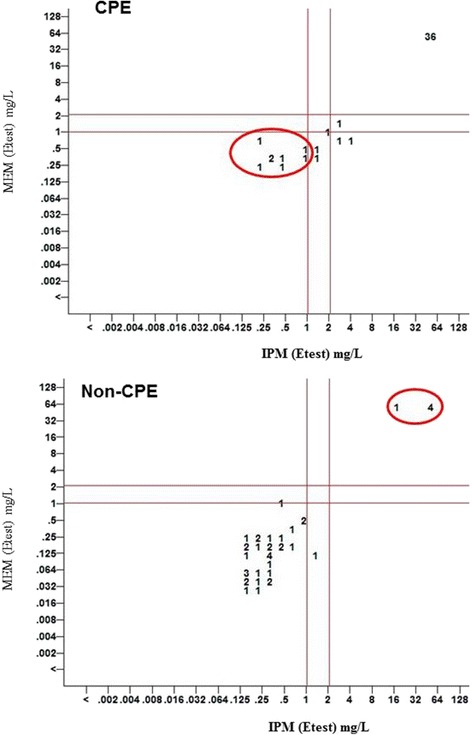
Scatter plot showing MIC of meropenem (MEM) Vs Imipenem (IPM). Note: Among Carbapenemase producing Enterobacteriaceae (CPE), 8 isolates with MEM and IPM MIC ≤1 mg/L (sensitive) were positive for carbapenemase gene. Among Non-carbapenemase producing Enterobacteriaceae (Non-CPE), 5 isolates with MEM and IPM MIC ≥ 16 mg/L (resistant) were negative for carbapenemase gene

Scatter plots (Fig. 2) showing MIC of meropenem and imipenem among Non-CPE (CRE isolates negative for carbapenemase genes, *n* = 41) and CPE (*n* = 50) were constructed. Majority of the Non-CPE isolates were susceptible to meropenem (0.047 mg/L - 1 mg/L) and imipenem (0.25 mg/L - 1 mg/L). However 5 isolates showed high level resistance to both imipenem and meropenem (MIC ≥ 32 mg/L) but were negative both by phenotypic test and PCR for carbapenemase genes, 1 isolate was susceptible to meropenem (MIC 0.125 mg/L) and resistant to imipenem (MIC 1.5 mg/L). Among CPE, 36 isolates showed high level resistance to both imipenem and meropenem. For one isolate meropenem and imipenem MIC were 1.5 mg/L and 3 mg/L respectively. 8 CPE isolates were susceptible to both imipenem (MIC range 0.38 mg/L – 1 mg/L) and meropenem (MIC range 0.38 mg/L – 0.75 mg/L), 5 isolates were susceptible to meropenem (MIC range 0.38 mg/L – 1 mg/L) but resistant to imipenem (MIC range 1.5 mg/L - 4 mg/L).

### Susceptibility patterns of CPE and MIC of colistin and tigecycline

Amongst 50 CPE, 27 and 13 isolates were resistant to 6 (amikacin, gentamicin, ampicillin, cefoxitin, ceftazidime, ciprofloxacin, ertapenem, imipenem) and 5 (ampicillin, cefoxitin, ceftazidime, ciprofloxacin, ertapenem, imipenem) classes of antimicrobial agents respectively.

**Fig. 3:**
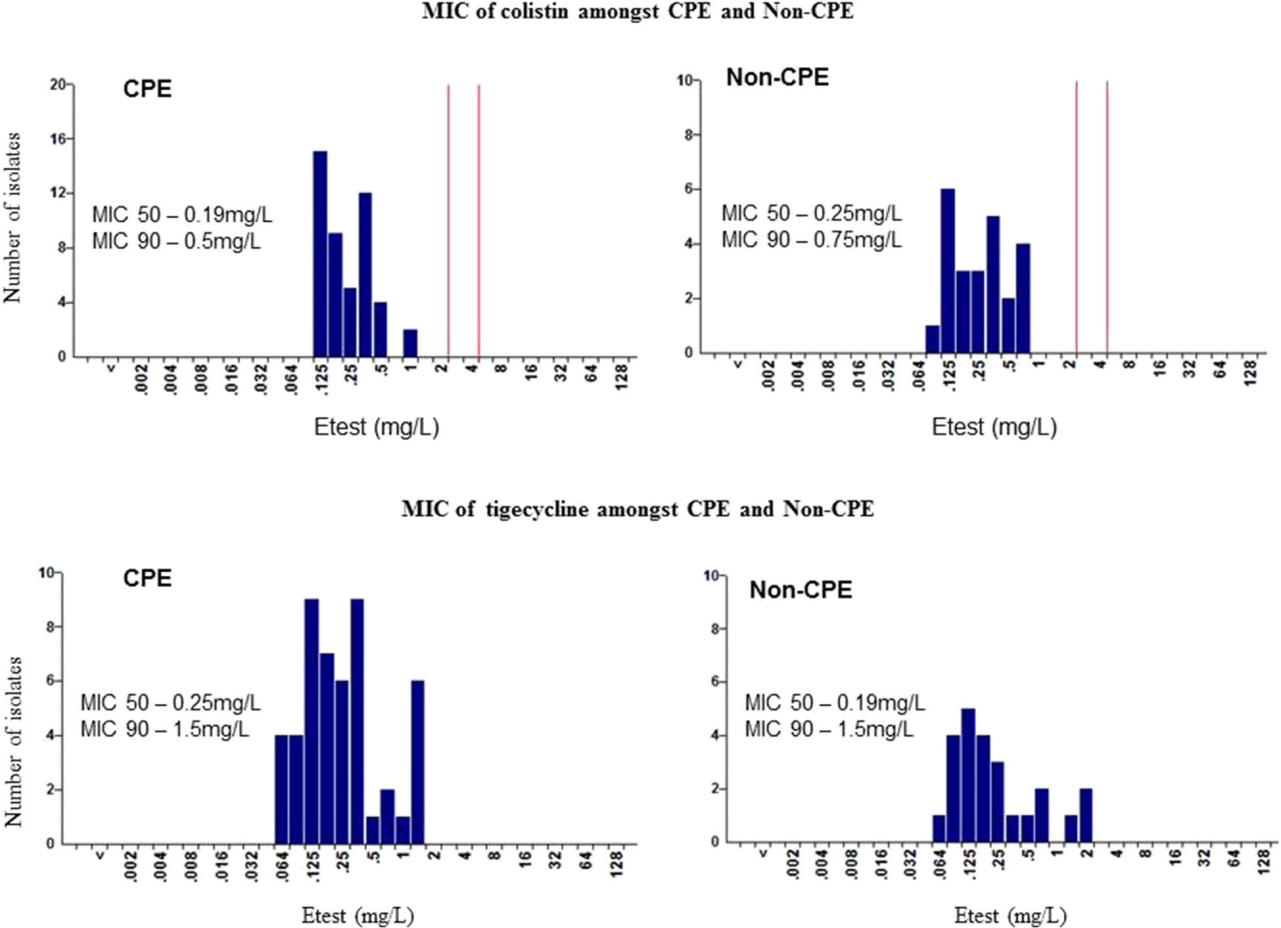
MIC of colistin and tigecycline amongst CPE and Non-CPE

MIC distributions to colistin and tigecycline among CPE and Non-CPE are shown in Fig. 3. All isolates were susceptible to colistin (MIC range 0.125 mg/L to 1 mg/L) and there was no significant difference (*p* value ≥0.05) in MIC50 and MIC90 among CPE and Non-CPE.

Using EUCAST 2011 breakpoint, the MIC range of tigecycline for CPE and Non-CPE isolates was 0.064 mg/L to 1.5 mg/L and 0.064 mg/L to 2 mg/L respectively. Although MIC50 and MIC90 did not differ, two Non-CPE isolates showed MIC at breakpoint i.e. 2 mg/L.

### Risk factors associated with CPE colonization in ICU patients (Table 3)

**Table 3:** Univariate analysis of risk factors for fecal colonization by CPE among ICU patients

RISK Factors	Total Patients *N* = 100			*P* value	Odds Ratio	Confidence Interval	
	No of patients	Patients with CPE (*n* = 26) (%)	Patients without CPE (*n* = 74) (%)				
Sex (Male)	58	17 (65.3)	41 (55.4)	0.37	1.52	0.600	3.849
Antibiotics use							
Carbapenems	14	7 (26.9)	7 (9.5)	0.027	3.528	1.1	11.304
Cephalosporins	45	13 (50)	32 (43.2)	0.55	1.313	0.536	3.125
Aminoglycosides	11	6 (23.1)	5 (6.8)	0.032	4.14	1.143	14.994
Augmentin	5	1 (3.8)	4 (5.4)	0.75	0.700	0.075	6.565
Fluoroqinolones	9	3 (11.5)	6 (8.1)	0.59	1.478	0.342	6.393
Interventions used							
Central venous line	35	12 (46.2)	23 (31.1)	0.16	1.901	0.761	4.744
Ventilator	61	21 (80.8)	40 (54.1)	0.01	3.570	1.216	10.483
Peripheral catheter	83	22 (84.6)	61 (82.4)	0.79	1.172	0.345	3.978
Naso-gastric tube	43	15 (57.7)	28 (37.8)	0.07	2.240	0.903	5.559
Oxygen Mask	21	6 (23.1)	15 (20.3)	0.7	1.180	0.403	3.454
Co-morbid conditions							
Surgery	15	7 (26.9)	8 (10.8)	0.048	3.039	0.976	9.461
Chronic lung disease	20	2 (7.7)	18 (24.3)	0.06	0.259	0.056	1.206
Neoplasia	7	1 (3.8)	6 (8.1)	0.4	0.453	0.052	3.955
Multivariate analysis							
Ventilator				0.027	0.291	0.097	0.871
Aminoglycosides				0.046	0.257	0.068	0.975

*CPE* carbapenemase producing Enterobacteriaceae

At the time of sampling, 59/100 (59 %) of ICU patients were under antibiotic therapy with 17 % receiving a combination of agents. The most frequently used antibiotics were third generation cephalosporin (*n* = 45) followed by carbapenems (*n* = 14), aminoglycosides (*n* = 11), fluroqinolones (*n* = 9), augmentin (*n* = 5). Invasive procedures among ICU patients included use of peripheral catheter (*n* = 83), ventilator (*n* = 61), naso-gastric tube (*n* = 43), central venous line (*n* = 35), oxygen mask (*n* = 21). Co-morbid conditions observed were chronic lung disease (*n* = 20), surgical procedure (*n* = 15) and neoplasia (*n* = 7). In Univariate logistic regression model, use of carbapenems (*p* value 0.02), aminoglycosides (*p* value 0.02), ventilator (*p* value 0.01) and surgical procedures (*p* value 0.048) were associated with a risk of CPE colonization. A multivariate logistic regression model was constructed to adjust for confounding variables. Use of aminoglycosides and ventilator were the only variables independently associated with CPE colonization. ICU stay was a significant risk factor as colonization was significantly higher on D4 of ICU stay as compared to ICU-D1 (*p* value 0.002, OR 8.633).

## Discussion

Very few studies have reported fecal carriage by CPE in non-outbreak conditions. A study from Pakistan [7] has reported fecal carriage of CPE (NDM-1) among 64/200 (18.5 %) hospitalized and out patients at military hospital Rawalpindi. As there are limited reports of gut colonization with CPE among ICU patients, this prospective study was undertaken to evaluate gut colonization with CPE and associated risk factors. Patients without prior history of hospitalization and admitted to the ICU for intensive care or ventilator support were included in the study. Colonization by CRE and CPE were studied at D1 and D4. Patients colonized with Enterobacteriaceae decreased from 63/100 on ICU-D1 to 58/100 on ICU-D4. This is explained by subsequent colonization with *Acinetobacter sp., Pseudomonas sp*., other nil-fermenters and *Candida sp*. by D4 of ICU stay (data not shown). The CPE colonization rate of 11 % on D1 was similar to colonization rates among non-hospitalized controls (6.6 %, *p* value >0.05). In view of our exclusion criteria these findings suggest acquisition of these isolates outside the hospital. This is further supported by NDM-1 being the predominant carbapenemase gene in both groups. NDM-1 producing Enterobacteriaceae may be disseminated in community and is supported by a recent study [6] which estimated a prevalence of 100 million carriers of NDM harboring bacteria in India alone. Unrecognized influx of NDM-1 producing Enterobacteriaceae in ICU may hinder infection control measures. It is thus critical to screen patients harbouring NDM-1 producing Enterobacteriaceae as they may be a source of infection acquired either endogenously or through horizontal transfer. In this study among 26 ICU patients colonized with CPE (DI and/or D4), 12 % developed sepsis due to NDM-1 producing Enterobacteriaceae. Interestingly although *Acinetobacter spp*. was the predominant cause of sepsis in ICU during the study period, we were also able to demonstrate NDM-1 gene among carbapenem resistant *Acinetobacter spp*. isolates suggesting the possibility of interspecies transmission (data not shown).

Patients colonized with CRE and CPE increased significantly from 20 % & 11 % on D1 to 35 % & 22 % respectively on D4 (*p* value <0.05). We could not study the transmission pathway of CPE among ICU patients due to non-availability of funds and resources. It is speculated that various mechanism could have contributed to the spread of CPE isolates from D1 to D4 of ICU stay. As carbapenemase gene co-exist with virulence and other antibiotic resistance gene on transmissible elements like plasmids, they can spread by horizontal transfer in diverse species in a highly populated ecosystem like gut. Secondly patients in ICU are exposed to the resistant flora, have co-morbid conditions and are subjected to invasive procedures, and hence these patients are at risk of acquiring MDR strain like VRE, ESBL, and CPE due to cross transmission resulting from breech of infection control practices. NDM-1 gene was the predominant carbapenemase gene observed among non-hospitalized patients and ICU patients on D1 of admission. Interestingly the diversity of genes increased on D4 of ICU stay and included NDM-1, OXA-48,181 and KPC. Moreover on D4 of ICU stay, 18 % (9/50) of the CPE isolates were found to have more than 1 carbapenemase gene. Similarly diversity of genera with carbapenemase genes was also observed. On D1, *E.coli* was the only Enterobacteriaceae with NDM-1 gene. On D4, NDM-1 gene was also isolated from *Klebsiella sp*., *Enterobacter sp*. and was found to co-exist with KPC and OXA genes suggesting horizontal transfer of plasmids and resistance genes among isolates. However the possibility of horizontal transfer of strains between patients via hands of health care workers cannot be ruled out.

There is paucity of data regarding risk factors predisposing to gut colonization with CPE. In this study, using multivariate analysis, ICU stays of >72 h, ventilator-use and treatment with aminoglycoside were independently associated with risk of CPE colonization. As mentioned above use of invasive procedure and ICU stay increase chances of transmission of multidrug resistant organism. Specific antibiotics and antibiotic classes have frequently been implicated as risk factors for colonisation or infection with CPE, and these include all the carbapenems, cephalosporins, fluoroquinolones, aminoglycosides and β-lactam/β-lactamase inhibitors [18]. The plasmids that confer such resistance frequently carry additional resistance determinants that confer cross-resistance to most other antibiotic classes. Consequently, prior use of any antibiotic may select for a carbapenemase-producing bacteria. Increased use of aminoglycoside in absence of carbapenem pressure can select resistance to carbapenems as was observed in present study. Similar to our findings, a study from Pakistan also reported that duration of hospitalization (irrespective of ICU) was statistically associated with a higher likelihood of carriage of CPE (*p* value < 0.05). However; this study reported co-amoxyclave as a risk factor of CPE colonization with NDM [19].

In present study, using 4 different in-house prepared selective media, carbapenemase gene was detected in 50 non duplicate isolates of *Escherichia coli, Klebsiella sp., Proteus sp.* and *Enterobacter sp*. The data on comparison on the performance of various media is not shown. There was an excellent correlation among the phenotypic test and PCR results for identification of CPE isolates. Diagnostic disks using meropenem ± boronic acid and meropenem ± dipicolinic acid were 100 % predictive of production of class A and class B enzymes respectively.

Forty one CRE were negative for various Carbapenemase genes screened (Non-CPE) and 5 of these isolates showed high level resistance to both meropenem and imipenem. As these isolates were positive for ESBL and/or AmpC, possibility of other coexisting mechanisms contributing to carbapenemase resistance either due to a novel carbapenemase gene or alteration in permeability due to over expression of efflux pump or alteration in outer membrane porins needs to be investigated.

Multidrug Resistance was common with resistance to 5 and 6 classes of antibiotics observed among CPE isolates from controls and ICU respectively, leaving few therapeutic options for treatment and includes colistin and tigecycline. All 50 isolates of CPE were susceptible for colistin and tigecycline with MIC range 0.125 mg/L -1 mg/L and 0.064 mg/L -1.5 mg/L respectively.

There are a few limitations of the study. Firstly, the data on co morbidities among controls which included non-hospitalized patients presenting at the out-patient department for routine stool examination as a part of health check was not available and hence the actual risk of healthcare associated colonization cannot be ruled out. Secondly, due to lack of resources we could not study the transmission pathway of CPE among ICU patients. However there is enough evidence of horizontal transfer of CPE among patients and diverse bacterial isolates.

## Conclusion

To conclude gut colonization by CPE among ICU patients on D1 is alarming, unrecognized influx of CPE in ICU may hinder infection control measure. NDM-1 carbapenemase was most common gene Exposure to ICU further increases the risk of colonization with diverse carbapenemase genes. Isolation of CPE with MIC to meropenem and imipenem within susceptible range (MIC ≤ 1 mg/L) further challenges their detection in surveillance cultures. Various other mechanisms may also contribute to carbapenem resistance. Therefore further characterization should be performed on all suspected isolates with slightest decrease in susceptibility to carbapenems especially ertapenem. Therapeutic options for treating carbapenem resistant Enterobacteriaceae infections include colistin and tigecycline.

## Abbreviations

CDC, centre for disease control; CLSI, clinical laboratory standard institute; CPE, carbapenemase producing Enterobacteriaceae; CRE, carbapenemase resistant Enterobacteriaceae; D1, day 1; D4, day 4; ICU, intensive care unit; ESBL, extended spectrum beta lactamases; EUCAST, European committee on antimicrobial susceptibility testing; MacI, MacConkey agar supplemented with imipenem at 1 mg/L; MacC ESBL, MacConkey agar supplemented with cefotaxime at 1 mg/L; MacD, MacConkey agar with standard imipenem, meropenem and ertapenem Disk; applied at the 4-, 8-, and 12-o’clock positions; MIC, minimum inhibitory concentration

## Additional file

Additional file 1: Proforma used for collection of data. (DOC 32 kb)

## References

[1] Andremont A (2003). Commensal flora may play key role in spreading antibiotic resistance. ASM News.

[2] Caprioli A, Busani L, Martel JL, Helmuth R (2000). Monitoring of antibiotic resistance in bacteria of animal origin: epidemiological and microbiological methodologies. Int J Antimicrob Agents.

[3] Grundmann H, Livermore DM, Giske CG, Cantón R, Rossolini GM, Campos J, Vatopoulos A, Gniadkowski M, Toth A, Pfeifer Y, Jarlier V, Carmeli Y. Carbapenem-non-susceptible Enterobacteriaceae in Europe: conclusions from a meeting of national experts. Euro Surveill. 2010;15(46). 10.2807/ese.15.46.19711-en 21144429

[4] Macpherson AJ, Harris NL (2004). Interactions between commensal intestinal bacteria and the immune system. Nat Rev Immunol.

[5] Kothari C, Gaind R, Singh LC, Chellani H, Saxena S, Deb M (2013). Community acquisition of β-lactamase producing Enterobacteriaceae in neonatal gut. BMC Microbiol.

[6] Walsh TR, Weeks J, Livermore DM, Toleman MA (2011). Dissemination of NDM-1 positive bacteria in the New Delhi environment and its implications for human health: an environmental point prevalence study. Lancet Infect Dis.

[7] Perry JD, Naqvi SH, Mirza IA, Alizai SA, Hussain A, Ghirardi S (2011). Prevalence of faecal carriage of Enterobacteriaceae with NDM-1 carbapenemase at military hospital in Pakistan, and evaluation of two chromogenic media. J Antimicrob Chemother.

[8] Gupta N, Limbago BM, Patel JB (2011). Carbapenem-Resistant Enterobacteriaceae: Epidemiology and Prevention. Clin Infect Dis.

[9] Guillet M, Bille E, Lecuyer H, Taieb F, Masse V, Lanternier F (2010). Epidemiology of patients harboring extended-spectrum beta-lactamase-producing Enterobacteriaceae (ESBLE), on admission. Med Mal Infect.

[10] Wiener J, Quinn JP, Bradford PA, Goering RV, Nathan C, Bush K (1999). Multiple antibiotic-resistant Klebsiella and Escherichia coli in nursing homes. JAMA.

[11] Laboratory protocol for detection of carbapenem-resistant or carbapenemase-producing, *Klebsiella spp*. and *E.coli* from rectal swabs. Centers for Disease Control and Prevention. 2008. http://www.cdc.gov/hai/pdfs/labsettings/klebsiella_or_ecoli.pdf. Accessed 6 Jul 2016.

[12] Winn WC, Allen SD, Janda WM, Koneman E, Procop G, Schreckenberger P, et al. Koneman’s color atlas and textbook of diagnostic microbiology. 6^th^ edition. Baltimore: Lippincott Williams Wilkins; 2006.

[13] Clinical and Laboratory Standard Institute (2012). Performance of standards for antimicrobial susceptibility testing; Twenty-first Information supplement M100–S22.

[14] Sambrook J, Fristch EF, Maniatis T. Molecular cloning: a laboratory manual. 2^nd^ edition. Cold Spring Harbor Laboratory Press; 1989.

[15] Woodford N, Fagan EJ, Ellington MJ (2006). Multiplex PCR for rapid detection of genes encoding CTX-M extended-spectrum (beta)-lactamases. J Antimicrob Chemother.

[16] Pérez-Pérez FJ, Hanson ND (2002). Detection of Plasmid-Mediated AmpC β-Lactamase Genes in Clinical Isolates by Using Multiplex PCR. J Clin Microbiol.

[17] Castanheira M, Deshpande LM, Mathai D (2011). Early dissemination of NDM-1 and OXA-181 producing Enterobacteriaceae in Indian hospitals: report from the sentry antimicrobial surveillance program, 2006-2007. Antimicrob Agents Chemother.

[18] Brink A, Coetzee J, Clay C, Corcoran C, Greune JV, Deetlefs JD, Nutt L, Feldman C, Richards G, Nordmann P, Poirel L (2012). The spread of carbapenem-resistant Enterobacteriaceae in South Africa: Risk factors for acquisition and prevention. S Afr Med J.

[19] Day KM, Ali S, Mirza IA, Sidjabat HE, Silvey A, Lanyon CV (2013). Prevalence and molecular characterization of Enterobacteriaceae producing NDM-1 carbapenemase at a military hospital in Pakistan and evaluation of two chromogenic media. Diagn Microbiol Infect Dis.

