# Supplementary material for: Risk factors for fecal carriage of carbapenemase producing Enterobacteriaceae among intensive care unit patients from a tertiary care center in India
Source: BMC Microbiol. 2016 Jul 8;16:138. doi: 10.1186/s12866-016-0763-y (PMC4938945; doi:10.1186/s12866-016-0763-y)
Supplement: Additional file 1: — Proforma used for collection of data. (DOC 32 kb) [file 12866_2016_763_MOESM1_ESM.doc]

**SUPPLEMENTARY FILE**

**Proforma used for collection of data**

| **Name** | **Age** | **Sex** | | **Date** | **Serial no** |
| --- | --- | --- | --- | --- | --- |
| ***DOA*** | ***MRD no*** | ***Ward*** | | ***Bed no*** | |
| **Clinical diagnosis** | | | |  | |
| **Date of Transfer to ICU** | | | **Date of discharge /outcome** | | |
| **Details of surgical procedure** | | | **Previous Hospitalization** | | |
| **Antibiotic History** | | |  | | |
| **Use of devices /Invasive procedures**  CVC  Urinary catheter  Tracheostomy  Ventilation  Peripheral Catheter  Nasogastric feeding  Shunt   Other specify | | | **Other morbid conditions**   Diabetes   Decubitus ulcer   Ischemic heart disease   Chronic lung disease   Chronic renal failure   Cerebrovascular disease   Neoplastic   Other specify | | |
| **Laboratory data** | | |  Growth on MacI   Growth on MaC ESBL   Growth on MaC D   Growth on TSB broth (CDC protocol) | | |
| **Phenotypic confirmatory test** | | |  Modified MHT   Synergy with DPA   Synergy with Boronic acid   Synergy with cloxacillin | | |
| **Organism**  **Antibiogram** | | | **MIC μg/ml**  Ertapenem  Meropenem  Imipenem  Tigecycline  Colistin | | |
| **Genotypic results** | | |  | | |
